# Management of Recurrent Stricture Formation after Transverse Vaginal Septum Excision

**DOI:** 10.1155/2015/975463

**Published:** 2015-05-11

**Authors:** Ridhima Gupta, Joseph D. Bozzay, David L. Williams, Robert T. DePond, Pickens A. Gantt

**Affiliations:** ^1^Department of Obstetrics and Gynecology, Charleston Area Medical Center, West Virginia University, Charleston Division, Charleston, WV 25302, USA; ^2^West Virginia University School of Medicine, Charleston Division, Charleston, WV 25304, USA

## Abstract

*Background*. A transverse vaginal septum (TVS) is a rare obstructing anomaly, caused due to improper fusion of Müllerian ducts and urogenital sinus during embryogenesis. *Case*. A 15-year-old girl presented with primary amenorrhea. She had multiple congenital anomalies. Initial examination and imaging investigation revealed the presence of a unicornuate uterus and a TVS. The TVS was excised; however the patient was unable to perform vaginal dilation postoperatively leading to recurrent stricture formation. She underwent multiple surgeries for excision of the stricture. The patient was eventually evaluated every day in the clinic until she was able to demonstrate successful vaginal dilatation in the presence of a clinician. *Summary and Conclusion*. Properly guided regular and intensive vaginal dilation after TVS excision may decrease the need of reoperations due to recurrent stricture formation.

## 1. Introduction

A vaginal septum is caused by incomplete fusion of the separating tissue between the fused Müllerian ducts and the vaginal plate [1]. The prevalence of this congenital abnormality is 1 per 30,000 to 84,000 women [1]. It occurs in any portion of the vagina and can be transverse, longitudinal, or oblique. The majority of vaginal septa are located in the upper and middle third of the vagina; and thicker septa usually are closer to the cervix [2]. The etiology of vaginal septa is unknown but may be multifactorial in origin, due to exposure to agents in utero, or associated with autosomal recessive inheritance [3]. Renal, musculoskeletal, and cardiac anomalies are rarely seen with transverse vaginal septa [1, 4, 5]. However, in one series, 20% of women with vaginal septa were diagnosed with renal defects [5].

In this report, we present a case of a thick TVS with concurrent unicornuate uterus and multiple congenital renal, skeletal, and cardiac abnormalities. The TVS was excised, but the postoperative course was complicated by recurrent stricture formation, which was subsequently managed by reconstructive surgery and vaginal dilatation.

## 2. Case Presentation

A 15-year-old girl presented with primary amenorrhea. She attained thelarche at 10 years of age and pubarche at 11 years of age. She had a history of cyclic pelvic pain since the age of 12. Her past medical history was remarkable for a ventricular septal defect as an infant which was subsequently repaired. She also had left renal agenesis which was diagnosed over a decade later. Multiple surgical procedures were performed for correction of severe scoliosis in her lumbar spine with placement of screws and rods. She had no family history of cardiac or genitourinary disease.

Physical examination revealed normal build with a height of 162 centimeters (cm). Her weight was 75 kilograms with a body mass index (BMI) of 28.6 kg/m^2^. She had normal development of secondary sexual characteristics, categorized as Tanner stage 5. Pelvic examination was limited since she was virginal.

**Figure 1:**
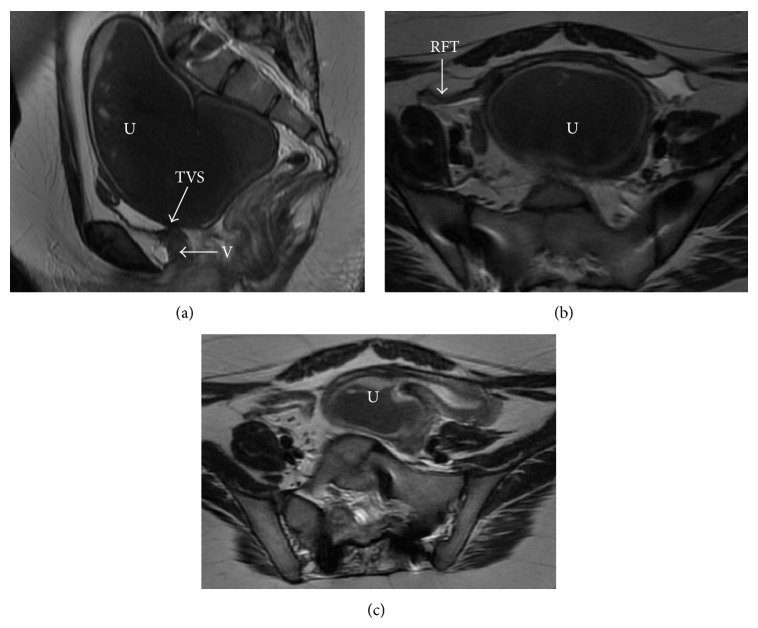
MRI of the pelvis, demonstrating site of transverse vaginal septum. (a) Sagittal T2 image, showing uterus distended with blood (U), the site of transverse vaginal septum (TVS), and narrowing at the level of vagina (V). (b) Axial T2 image, demonstrating distended uterus (U). (c) Axial T2 image, showing the distended left unicornuate uterus. U: uterus, TVS: transverse vaginal septum, V: vagina, and RFT: right fallopian tube.

On laboratory analysis of her blood, the complete blood count and biochemistry were within the normal range. Endocrinological laboratory values were also within normal limits and consistent with her age, including thyroid stimulating hormone of 3.0 uU/mL, testosterone of 11 ng/dL, and hemoglobin A1C of 5.3%. A pelvic transvaginal sonogram suggested that the uterus was distended with fluid. Magnetic resonance imaging (MRI) showed a 12 × 7 cm fluid collection in the uterus and an obstruction likely at the level of the vagina which was concerning for imperforate hymen or a transverse vaginal septum. The MRI also showed marked underdevelopment of the right side of the uterus (Figure 1). No other Müllerian anomalies were identified.

**Figure 2:**
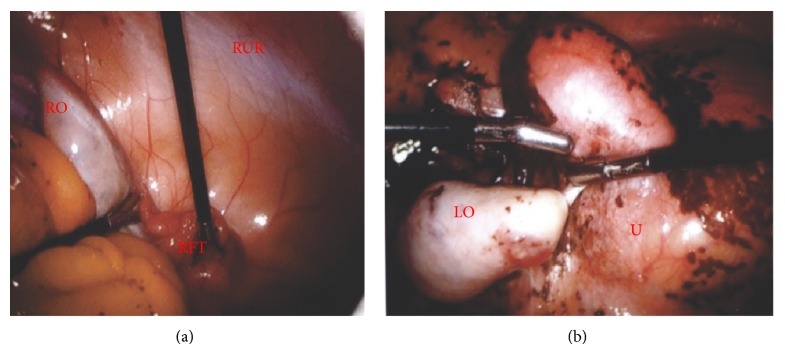
Laparoscopy of the pelvis. (a) Right fallopian tube (RFT), right ovary (RO), and a right uterine remnant (RUR). (b) Left ovary (LO) and distended uterus (U) with hemosiderin (brown).

**Figure 3:**
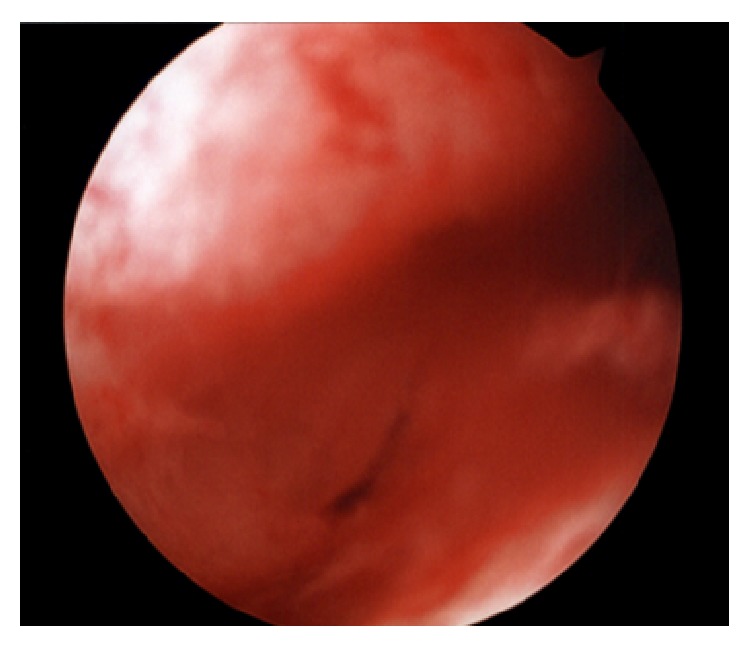
Vaginoscopy: stenosis of vaginal septum site, three months after the initial excision and reapproximation.

The patient was diagnosed with multiple Müllerian anomalies as the cause of her primary amenorrhea. Surgical intervention was suggested to the patient and her family. Exam under anesthesia revealed a 3 cm blind ending vagina and a 7 × 7 cm pelvic mass. She subsequently underwent diagnostic laparoscopy (Figure 2) and vaginoscopy (Figure 3). Intraoperatively she was found to have a distended left unicornuate uterus with a right uterine horn with associated fallopian tube on the right side. Bilateral ovaries and fallopian tubes were present. Extensive hemosiderin deposits were identified in the pelvic cavity. On local genital examination, a 1 cm thick complete TVS was identified at the junction of the upper one-third and the lower two-thirds of the vagina. This TVS was diagnosed under ultrasound (US) guidance. The septum was grasped with Allis tissue holding forceps and a stab incision was made. The hematocolpos was drained, and her cervix was subsequently visualized. The vaginal septum was then excised circumferentially. The vaginal epithelium was then approximated with polyglactin 910 throughout its circumference using simple end-to-end anastomosis. A sponge vaginal mold coated with estrogen cream was then placed in vagina to prevent stricture formation.

She was discharged home on postoperative day one. The vaginal mold was removed on the day of discharge and the patient was advised to apply estrogen cream in the vagina locally. As our patient was not sexually active, she was instructed to manually dilate the vagina everyday with the placement of dilators. She was given 10 mg of norethindrone daily for 4 weeks to prevent her menstrual cycle and allow adequate healing of her surgical site. After withdrawal of norethindrone, she had a normal menstrual period.

Two months later at a follow-up visit she presented with pelvic discomfort and dark brown discharge from her vagina. Clinical examination revealed a stricture at the surgical site resulting in stenosis of the vagina. This stricture was then dilated under anesthesia. The margins of the stricture were surgically resected and the vaginal epithelium was approximated. She was discharged home on postoperative day two. She was again extensively counseled regarding the use of manual vaginal dilation methods in order to prevent recurrent stenosis.

However, she was unable to perform manual vaginal dilatation at home due to vaginal spasms and apprehension. She presented again a month later with vaginal stenosis. The stenosed vaginal edges were again excised and reapproximated and a plastic vaginal stent was placed in the vaginal canal to maintain patency. This stent was then removed after two months and she was again educated about the dilatation methods, but she still presented with restenosis three weeks later. The stenosed margins were resected again and the patient was then evaluated every day in the clinic where she demonstrated vaginal dilatation in the presence of a clinician. After 3 months, the patient was able to perform vaginal dilatation successfully without any apprehension and the vaginal patency was maintained without any recurrent stricture formation. She was also instructed to place a vaginal mold nightly for the next six months. Subsequent evaluation at 4 and 6 months from the last surgery demonstrated a patent vaginal canal with adequate vaginal length of 8 cm. She was then experiencing normal menstrual cycles.

## 3. Discussion

Treatment of a TVS depends on the patient. Vaginal dilators are the preferred nonsurgical choice for patients with small septa [5]. Dilation techniques may be used in lieu of surgery, before surgery in order to improve outcomes, and after surgery to prevent strictures, scarring, or stenosis of the surgical site [5].

If the transverse vaginal septum cannot be treated with vaginal dilation alone, then surgery offers a permanent solution. Small transverse vaginal septa (<1 cm in thickness) may be treated by excision with a simple end-to-end anastomosis of the vaginal epithelium or a Z-plasty [2, 3, 5]. Larger septa (>1 cm) may require preoperative vaginal dilation followed by a longitudinal Z-plasty technique to reduce stenosis and contraction of scarring at the site [3, 5]. The Olbert balloon catheter technique has also been described to maximize the vaginal mucosa available for anastomosis and avoid postoperative narrowing of the vagina [6]. However, in our patient, the high location of her TVS limited us to utilize this technique.

Postoperative vaginal dilation or a vaginal mold may help in decreasing the scarring and stenosis of the surgical site [3, 5]. Postoperative vaginal dilation is critical to the success of the procedure. Our patient was apprehensive about performing vaginal dilation, which is not uncommon in adolescents [5]. After many attempts to help our patient become comfortable using dilators, her inability to perform vaginal dilatation caused multiple episodes of restenosis and necessary surgical resections. Similar to our report, Joki-Erkkilä and Heinonen reported 2 out of 3 patients needed reexcision of the stricture, which formed after excision of a complete TVS [7]. Even after leaving the plastic vaginal stent in the vagina of our patient for two months, the site restenosed after the stent was removed. Wierrani et al. reported success with patients of age 15.4 ± 2.8 years using a mold for 5–8 months postoperatively and then a nightly mold for 6 months afterwards if the patient was not sexually active [3]. Our patient was managed surgically with end-to-end anastomosis of the vaginal epithelium after excision of the septum, which is very similar to the technique described by Wierrani et al. However, as exemplified by our patient, inadequate vaginal dilatation postoperatively can lead to stricture formation. Therefore, these patients may be better served with a long-term mold or stent.

Daily vaginal dilation needed after TVS excision at such a young age can be challenging to the patient. This case was unique in that success was achieved only after daily guidance through the use of vaginal dilators. In our patient certain barriers were identified as responsible for recurrent stricture formation including her young age, apprehension to insert the vaginal mold, and a certain degree of vaginismus that was experienced by her at the time of insertion of the vaginal mold. The patient's mother was also instructed on the use of vaginal dilators so that she would be able to assist our patient in using the vaginal mold, but no success was achieved. The decision was then made to evaluate the patient every day in clinic where she demonstrated the vaginal dilatation in presence of the clinician. She was psychologically desensitized to perform dilatation and her apprehension resolved. Ultimately, she developed confidence in performing vaginal dilatation. As the patient underwent multiple operative procedures to resect the stricture the importance of vaginal dilation method cannot be overemphasized. We were also concerned initially about the risk of vaginal shortening as our patient underwent multiple surgical resections; however with the regular vaginal dilatation, our patient now has an adequate vaginal length.

It is important to follow up with these patients in the long term as they may experience adverse outcomes after the surgery. Dyspareunia, menstrual irregularities, fertility complications, and preterm labor are not uncommon [2, 5]. Recognition and intervention at younger ages by draining the accumulated blood and possibly preventing endometriosis may preserve fertility [2, 4]. Rock et al. reported that patients were less likely to conceive after surgical correction if their transverse vaginal septum was located in the upper or middle third of the vagina as was true in our patient. If they did succeed, 50% of pregnancies ended in spontaneous abortions [4]. Patients should be educated about these potential long-term complications and the need to closely follow up with their provider when trying to become pregnant.

**Figure 4:**
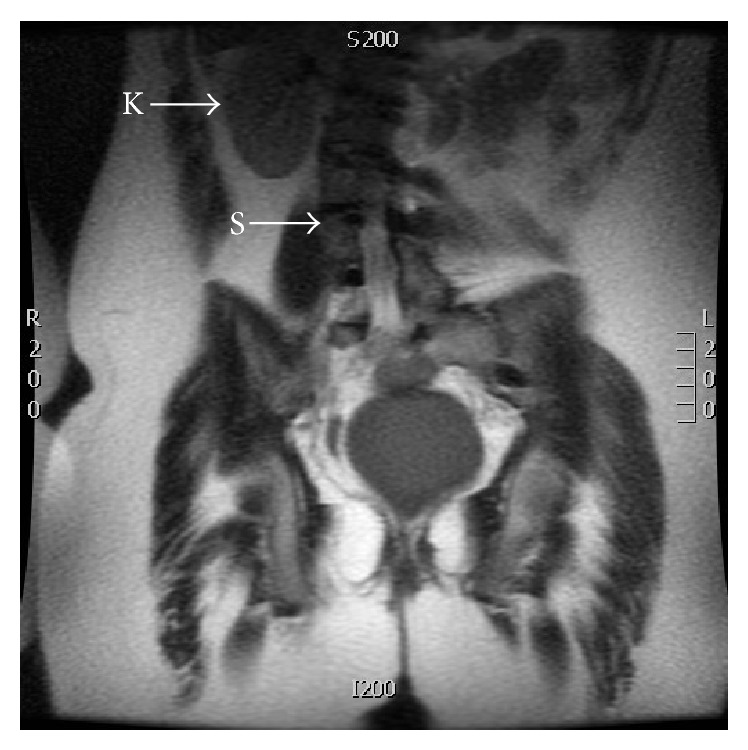
MRI of the lower abdomen and pelvis, demonstrating single kidney and scoliosis. Coronal T2 image, showing right Kidney (K) only with absence of left kidney and severe scoliosis (S).

This case also presents a unique constellation of multiple rare congenital malformations, including a transverse vaginal septum, unicornuate uterus, left renal agenesis contralateral to the uterine remnant, ventricular septal defect, and severe scoliosis (Figure 4). Based on a systematic literature search using MEDLINE (Ovid and PubMed) databases from 1950 to March 2015 using keywords “unicornuate uterus, renal agenesis and transverse vaginal septum” and “genitourinary congenital malformations” with no limits placed, no similar case reports were identified demonstrating concomitant presence of unicornuate uterus with transverse vaginal septum and renal agenesis contralateral to the uterine remnant. Renal agenesis has been described in the literature as occurring ipsilateral to the uterine remnant obviously due to the interaction of the embryonic ducts on the same side. More investigations are needed to examine the underlying genetic basis of this association. Genetic counseling and evaluation were offered to our patient, but she and her family declined.

Our report demonstrates successful surgical management of a transverse vaginal septum and the complications that can occur at the surgical site. It illustrates the importance of monitoring a vaginal septum resection site for restenosis and the unique management necessary when a young patient is apprehensive about vaginal dilatation. Vaginal dilatation is crucial after excision of TVS. Long-term follow-up is important for the surgical success and potential issues that the patient may encounter throughout her life.

## References

[1] Caloia D. V., Morris H., Rahmani M. R. (1998). Congenital transverse vaginal septum: vaginal hydrosonographic diagnosis. *Journal of Ultrasound in Medicine*.

[2] Deligeoroglou E., Iavazzo C., Sofoudis C., Kalampokas T., Creatsas G. (2012). Management of hematocolpos in adolescents with transverse vaginal septum. *Archives of Gynecology and Obstetrics*.

[3] Wierrani F., Bodner K., Spängler B., Grünberger W. (2003). ‘Z’-plasty of the transverse vaginal septum using Garcia's procedure and the Grünberger modification. *Fertility and Sterility*.

[4] Rock J. A., Zacur H. A., Dlugi A. M., Jones H. W., TeLinde R. W. (1982). Pregnancy success following surgical correction of imperforate hymen and complete transverse vaginal septum. *Obstetrics and Gynecology*.

[5] Lankford J. C., Mancuso P., Appel R. (2013). Congenital reproductive abnormalities. *Journal of Midwifery and Women's Health*.

[6] Layman L. C., McDonough P. G. (2010). Management of transverse vaginal septum using the Olbert balloon catheter to mobilize the proximal vaginal mucosa and facilitate low anastomosis. *Fertility and Sterility*.

[7] Joki-Erkkilä M. M., Heinonen P. K. (2003). Presenting and long-term clinical implications and fecundity in females with obstructing vaginal malformations. *Journal of Pediatric and Adolescent Gynecology*.

